# Heterozygous Alpha-1 Antitrypsin Deficiency Causing Pulmonary Emboli and Pulmonary Bullae

**DOI:** 10.7759/cureus.14759

**Published:** 2021-04-29

**Authors:** Toufic Tannous, Claudia Rosso, Matthew Keating

**Affiliations:** 1 Internal Medicine, Roger Williams Medical Center/Boston University, Providence, USA; 2 Internal Medicine, University of California Irvine, Irvine, USA; 3 Hematology and Oncology, University of California Irvine, Irvine, USA

**Keywords:** alpha-1 antitrypsin, pulmonary embolism (pe), pulmonary bulla

## Abstract

Alpha-1 antitrypsin deficiency is an autosomal co-dominant disease known for different genetic alterations in the serine protease inhibitor enzyme by which different disease phenotypes can manifest. The lung and the liver are the most common organs involved. The severity of the disease depends on the phenotypes involved. However, emerging evidence shows that this disease can impact multiple organ systems and may even develop regardless of the phenotype. We describe a case of a young man with a known history of the MS phenotype who presented with chest pain and was found to have pulmonary emboli and bullae. His past medical history was relevant for a gastric ulcer and elevated liver enzymes. Due to this young man’s age and lack of risk factors for the aforementioned diseases, we propose that these findings were manifestations of his MS phenotype. This case raises multiple questions challenging the presumed benign nature of the MS phenotype. We propose a closer follow-up and lower threshold for diagnostic studies in patients with the heterozygous form.

## Introduction

Alpha-1 antitrypsin (AAT) belongs to the family of ‘SERPIN’ proteins or serine protease inhibitors. This protein is responsible for regulating neutrophil elastase, an enzyme released by neutrophils and macrophages to destroy bacteria. In the absence of AAT, neutrophil elastase can also destroy native lung tissue, leading to emphysema. AAT deficiency (AATD) is known to encompass a spectrum of diseases, most notably affecting the lung and liver. Laurell and Eriksson first described AATD in 1963 when identifying an association of low serum levels of AAT with emphysema [1]. With the help of gel electrophoresis, multiple phenotypes of AATD have since been described. In fact, more than 100 allele deficiencies have been found so far [2].

AATD is an autosomal co-dominant disease. Some authors describe the genetic inheritance pattern as autosomal recessive; however, more evidence is accumulating to link clinically significant diseases to the heterozygous disease phenotypes, hence the co-dominant designation. The genetic variants are mostly attributed to a single amino acid substitution within protein subtypes. They are classified based on their mobility on an acid starch gel. For example, F=fast, M=medium, S=slow, and Z=very slow. The first step in diagnosing the disease is by measuring the AAT level in the serum. The ZZ form has the lowest concentrations, whereas MM has normal concentrations. The MM phenotype indicates homozygosity for the wildtype allele, which, in turn, translates into normal levels of AAT protein production. Patients with two Z alleles typically have AAT levels around 15% of normal, whereas patients with two S alleles typically have AAT levels around 60% of normal [3]. MZ is known for intermediate concentrations of AAT and is considered the heterozygous disease phenotype. MS typically has concentrations near the low end of normal. The MZ and MS phenotypes were initially described as carrier states, but this fails to acknowledge emerging clinical manifestations with these phenotypes.

AATD patients most commonly present with emphysema or liver disease. Most of the patients in these cases possess the ZZ phenotype and, in rare situations, the MZ and MS phenotype. The emphysema tends to have a basilar pattern but has also been well described in the apical region and is sometimes complicated by the formation of pulmonary bullae. A full spectrum of liver disease including cirrhosis has also been described. There are also reports identifying associations with hepatocellular carcinoma, peptic ulcers, panniculitis, coagulopathy, and pulmonary emboli. In one case of a ZZ homozygote, a patient was found to have recurrent pulmonary emboli [4].

The MS phenotype is known for intermediate concentrates of serum AAT. The S phenotype is formed when glutamic acid is substituted by valine at position 264. The MZ phenotype has been traditionally thought of as the more severe heterozygous AATD phenotype versus the MS phenotype. To our knowledge, there have been no published cases describing the coexistence of pulmonary emboli and bullae in the setting of an AATD heterozygous MS phenotype.

## Case presentation

**Figure 1:**
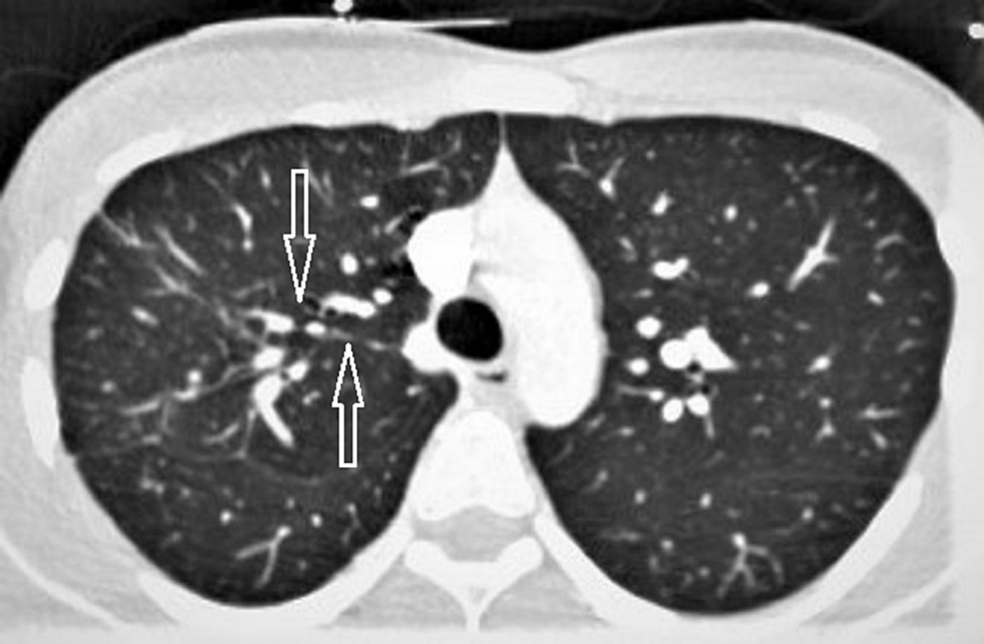
CTA showing the pulmonary emboli in the right upper lobe apical segmental and subsegmental branch of the right pulmonary artery (arrows)

**Figure 2:**
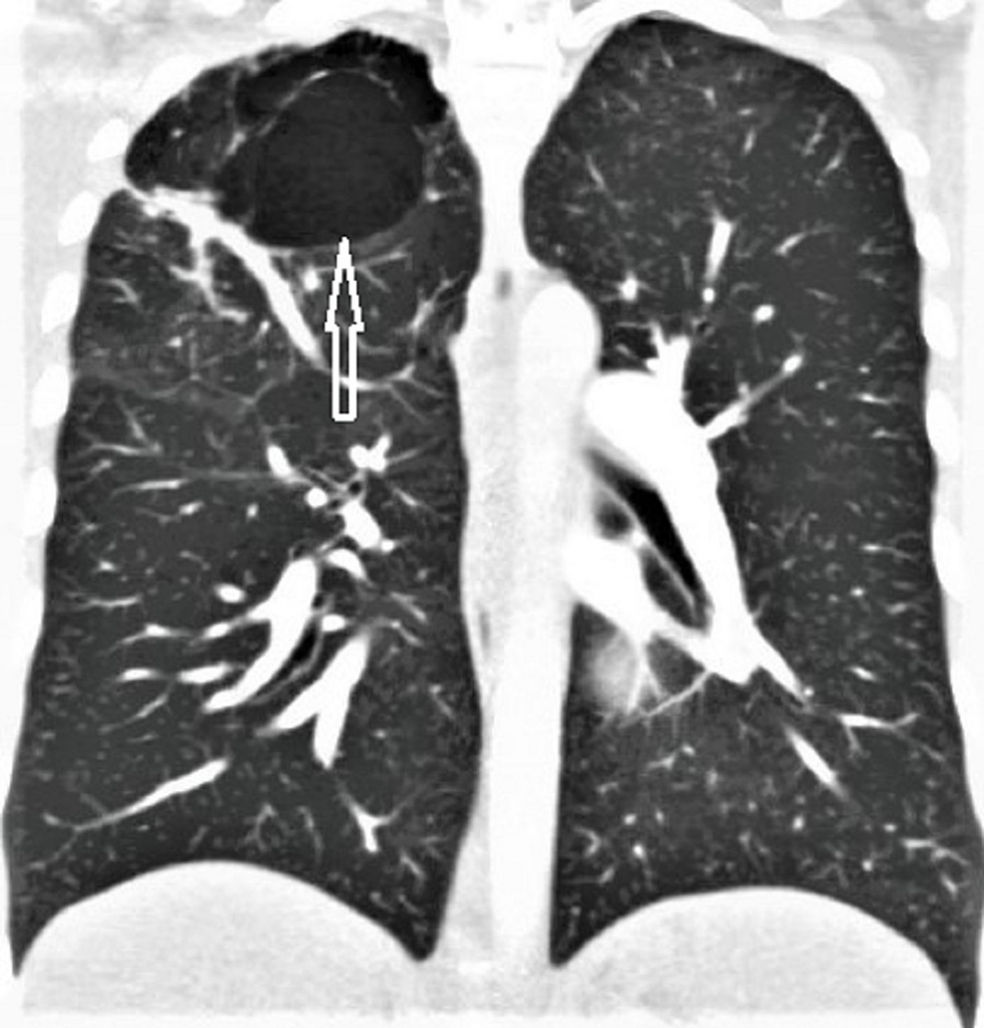
CTA showing one of multiple large bullae in the right upper lobe with associated pleuro-parenchymal scarring (arrow)

A 22-year-old patient with a past medical history of elevated liver enzymes and gastric antrum erosion identified three years ago presented to the emergency department with complaints of chest pain and shortness of breath. Physical examination, lab workup, and an EKG were unremarkable. Despite the patient’s lack of risk factors, a computed tomography angiography (CTA) of the chest was performed to rule out a pulmonary embolism (PE). Results revealed a pulmonary emboli in the right upper lobe apical segmental and subsegmental pulmonary artery, as well as multiple large bullae in the right upper lobe with associated pleuro-parenchymal scarring (Figures 1, 2). He was admitted to the hospital and started on a heparin drip. After his symptoms improved, he was discharged on apixaban and instructed to follow up with a pulmonologist and hematologist.

On outpatient hematology clinic follow-up, he was tolerating apixaban administration well without complications aside from occasional minor nosebleeds. He denied cigarette or marijuana smoking history. He denied recreational or IV drug use. He was a full-time college student who was studying anthropology. There was no recent travel history outside of the continental United States. He used to compete in ultra-marathons and remained very active with exercise. He denied any history of tuberculosis or COVID-19 or recent known exposures. He denied any history of lung infections. He previously tested negative for HIV. He denied any personal or family history of blood clots, Marfan’s syndrome, Ehler’s Danlos, sarcoidosis, or Sjogren’s syndrome. He denied previous hospitalizations or premature birth. Looking back at the patient’s liver function test (LFT) workup three years prior, it was discovered that he had tested positive for heterozygous AATD, the MS phenotype. Serum AAT level was determined to be 111 mg/dL (normal: 100-240 mg/dL) by AAT phenotyping, and the AAT level was confirmed on repeat testing. Laboratory workup was pertinent for aspartate aminotransferase level (AST) of 74 U/L (normal: 5-40 U/L) and alanine aminotransferase level (ALT) of 45 U/L (normal: 7-55 U/L) and was unremarkable for the following: hepatitis B, hepatitis C, HIV, alfa-fetoprotein, anti-smooth muscle antibody, antinuclear antibody, ceruloplasmin, and quantitative immunoglobulins. He also had a gastric antrum erosion diagnosed that same year via endoscopy.

He was instructed to complete a three- to six-month course of apixaban. Clinical reassessment at a three-month interval was planned with interim pulmonary function test studies and consultation with a pulmonologist for his lung findings in the setting of AATD. A hypercoagulable workup was planned upon completion of his apixaban course seeing as many of the results, including protein C, protein S, and antithrombin levels, would have been impacted by apixaban administration.

## Discussion

Many diseases have been described in the setting of AATD. The organs most affected include the liver and the lungs. However, other diseases have been linked to AATD, such as anti-neutrophil cytoplasmic antibodies (ANCA) vasculitis, rheumatoid arthritis, inflammatory bowel disease (IBD), aneurysms, and panniculitis. In the majority of cases, the ZZ phenotype is the culprit behind these findings. The MS phenotype, on the other hand, has not previously been linked to the aforementioned diseases despite being present in an estimated 4.46% of the population in the United States [5]. In our case, the patient was known to have the MS phenotype of AATD. This was discovered when the patient was first diagnosed with a gastric antrum erosion and elevated liver enzymes. Workup for liver diseases then came back positive for AATD, the MS phenotype. Three years later, he presented with chest pain and was found to have right upper lobe bullae as well as pulmonary emboli.

When it comes to lung diseases, AATD is well known to cause emphysema, however, with mixed evidence for an association of emphysema with the MS phenotype. The deficiency of AAT causes an imbalance favoring neutrophil elastase, which results in a panacinar form of emphysema. When it comes to the intermediate phenotypes, MZ and MS, such cases of lung involvement are rare. Those cases of emphysema that were reported were seen in the context of the MZ type, and certain associations with race (white and African American) as well as with smoking history have been identified [6]. What makes our case interesting is that we are reporting this finding in the MS phenotype. Data on an association between the MS phenotype and emphysema risk have been mixed to date. Dahl and Nordestgaard showed in a meta-analysis of 17 studies of MS patients that there was an odds ratio of 1.2 for these patients to develop chronic obstructive pulmonary disease relative to the control population [7]. Case-control studies have shown evidence both for and against an association [8-10].

In AATD, emphysema tends to affect the lung bases more often than the apical region [11]. The areas of emphysema can be complicated by the formation of lung bullae, particularly in severe AATD [12]. A few cases of bullous emphysema involving the lung bases have been described with the MZ phenotype [13]. One case of apical bullous emphysema has also been described with the MZ phenotype [14]. Our case is the first reported instance to our knowledge of bullous emphysema in an MS phenotype, and the apical distribution makes the finding even more unusual.

AATD has also been linked to certain vessel diseases such as aneurysms and ANCA-positive vasculitis, and there is slowly emerging evidence of an association with coagulopathy. Reports describing clot formation in the setting of AATD are scarce. Most of these reports describe pulmonary emboli in the setting of a patient with the ZZ phenotype. In one case, the patient presented with recurrent emboli [4]. Tanash et al. identified a seven-fold higher incidence of PE in severe AATD patients compared with the general population [15]. There are a couple of theories behind the coagulopathy of AATD: unopposed activity of plasminogen activator, which, in turn, activates the coagulation cascade, and unopposed proteinase-3 activity, which leads to endothelial cell damage/activation [4,16]. What is unclear in our case is whether the pulmonary clot was formed due to pressure applied by the expanding bullae on the artery or due to an underlying coagulopathy with the AATD. For pulmonary bullae to be causative of the pulmonary clot, the bullae would need to apply pressure over the artery to cause a state of blood stasis and thus potentially form a clot. However, this would mean that the bullae are near the vessel or at least in its immediate vicinity. A review of the imaging shows that the clot and the bullae, while in the same lobe, are anatomically distant. At this point, whether the AATD or some other undetermined cause is responsible for the PE is unknown. In recent years, the number of AATD-associated diseases reported in the literature has increased. Pulmonary emboli may be yet another association to add to this list. If so, this finding in the setting of the MS phenotype makes it more unique.

We are hoping that this case may shed light on some of the unprovoked pulmonary emboli that occur in the younger generation, with no apparent risk factor or known medical condition. One prospective observational study reported that more than 30% of pulmonary emboli are idiopathic [17]. Whether it is necessary to check for the MS phenotype or any other phenotype of the AATD spectrum during an unprovoked embolus workup remains unclear. However, our patient’s circumstances may indicate that in some cases of concomitant lung bullae and pulmonary emboli, a workup for AATD would be very reasonable.

Liver disease is a very common manifestation of AATD. It is proposed that the cause of hepatocyte injury is distinct from the mechanism in which the lungs are injured. We mentioned earlier that in emphysema, the imbalance between elastase and AAT would lead to uncontrolled, elastase-provoked damage. However, in some phenotypes with liver disease, the quantity of AAT has been found to be in the normal range. Therefore, the reason the hepatocytes are damaged is not due to the direct elastase effect in the setting of deficient AAT but rather due to the accumulation of defective AAT in the hepatocyte endoplasmic reticulum that eventually leads to cell necrosis [18]. This pattern of liver damage is mostly seen in the ZZ phenotype. Our patient was found to have elevated liver enzymes; however, there were no stigmata of liver disease yet. Based on the workup performed, the only association that can explain the elevated liver function tests is the presence of the MS phenotype of AATD. According to our literature review, such cases have not been described frequently. However, this would certainly trigger a need for constant follow-up on the patient’s liver status. Given the current information available on our patient, it would be implausible to conclude that his pulmonary embolus is secondary to liver damage and cirrhosis, as the patient’s liver function tests, clinical exam and radiological findings steer us away from the presence of a defective liver.

Some cases of AATD have been linked to gastrointestinal (GI) disorders, especially IBD. It is yet unclear whether the deficiency in AAT causes an unopposed proteolytic elastase activity that causes damage to the bowel wall directly or damage to the vessel wall that supplies the area. There have also been a few cases of peptic ulcer disease in the setting of AATD. Early onset peptic ulcer disease in young adults has been linked to decreased AAT levels and AATD, with the association extending to heterozygous AATD as well as to first-degree relatives [19,20]. In our patient’s situation, he was first referred for an upper endoscopy due to persistent heartburn and upper GI discomfort, prompting the finding of a gastric antrum erosion. For a healthy patient who is a non-smoker and does not take any medications, this is certainly not a common finding. It would be interesting to further link AATD with this diagnosis, especially in the case of an MS phenotype; however, more studies are needed to prove this association.

The findings of a gastric antrum erosion, pulmonary embolus, and bullae in an AATD MS phenotype are certainly unique. Most patients with the MS phenotype go undiagnosed as they rarely develop any clinical symptoms. In our case, multiple findings have surfaced and they are unusual in patients with the MS phenotype. Gupta et al. described a mutation of the AAT glycoprotein whereby arginine replaces methionine in the enzyme active center, rendering the protein into a coagulation pathway protease inhibitor. The consequence of this is a life-threatening hemorrhagic disease [4]. This begs the question: Can a different form of mutation create a fibrinolytic pathway protease inhibitor? What does this mean moving forward? For our patient, laboratory and imaging follow-up will be required to check on his liver status. There will be a risk for pneumothorax moving forward, and pulmonary follow-up is required. Anticoagulation will be continued; however, duration and discontinuation will need to be revisited. Referral for genetic counseling will be recommended. This case describes multiple findings in a healthy young man with no risk factors. These findings are potentially associated with AATD and thereby bring the MS phenotype into the spotlight.

## Conclusions

AATD can be linked to multiple clinical findings, especially those involving the lung and liver. The most notorious phenotype is the ZZ phenotype, which has been shown to be associated with most of the AATD disease manifestations. Based on the literature, the MS phenotype is known for having more of a benign nature, and the few diseases linked to it are described in some case reports. Our case describes multiple life-threatening findings in a patient with the MS type. This, in our opinion, should trigger a closer look into patients who have been found to have this phenotype or at least prompt consideration of AATD testing in similar situations. Although often clinically silent, some manifestations of the MS phenotype could appear early on and in an undesirable form.
